# A96 RAPID IMPLEMENTATION OF AN EVIDENCE-BASED, VIRTUAL COVID-19 VACCINE EDUCATION CLINIC AT NOVA SCOTIA COLLABORATIVE INFLAMMATORY DISEASE CLINIC (NSCIBD)

**DOI:** 10.1093/jcag/gwab049.095

**Published:** 2022-02-21

**Authors:** H Komeylian, J Jones, M Stewart, C Heisler, K Phalen-Kelly, B Currie

**Affiliations:** 1 Digestive Health and Endoscopy, Dalhousie University, Halifax, NS, Canada; 2 Medicine, Dalhousie University, Halifax, NS, Canada; 4 Gastroenterology, Research Services, QEII Health Sciences Centre, Halifax, NS, Canada; 5 QEII Health Sciences Centre, Halifax, NS, Canada

## Abstract

**Background:**

Rapid adaptation of clinical management as well as policy decisions in relation to implementation of COVID-19 vaccination programs for persons living with IBD has been required throughout the pandemic.

**Aims:**

To meet the need for public health-mandated COVID-19 vaccine education for patients living with IBD in Nova Scotia a novel, evidence-based, virtual COVID-19 vaccine educational intervention was developed, implemented, and evaluated.

**Methods:**

An observational, cross sectional, implementation-effectiveness study was conducted at the NSCIBD program between April and July, 2021. The educational intervention consisted of a standardized evidence-based letter describing risks and benefits of COVID-19 vaccine emailed to patients in advance of a virtual clinic appointment. Virtual appointments were offered to all patients contacting the NSCIBD program with questions or concerns about vaccination. During these virtual visits standardized, evidence-based information was provided by a gastroenterologist (n=2) or IBD nurse practitioners (n=2) and patients were provided with an opportunity to address specific disease and treatment related concerns. Following the session, a link to an anonymous questionnaire was distributed via email to evaluate key implementation metrics including satisfaction, appropriateness, usefulness, perceived impact on knowledge and vaccine hesitancy, and recommendations for improvement. Data analysis was descriptive. Group means were expressed as proportions for categorical variables and means for numerical variables.

**Results:**

A total of 298 patients participated in a virtual patient education session of which 265 provided a valid email address and invited to participate in the on-line survey. The response rate was 49% (131/265). Before the session, 48.9% (64/131) expressed vaccine hesitancy. Twenty-six percent (35/131) expressed concerns relating to risks versus benefits of COVID-19 vaccines. Ninety-one percent (119/131) of respondents found the education program helpful. The proportion of those willing to get vaccinated rose from 61% (pre) to 86.3% (post). Only 1.5% (2/131) indicated they would not get vaccinated. Seventy-seven percent (101/131) found the written and virtually administered educational content to be satisfactory. Eighty-eight percent (115/131) of respondents were willing to participate in similar types of virtual education offerings in the future.

**Conclusions:**

Implementation of an evidence-based, multidisciplinary, virtual COVID-19 vaccination education intervention was perceived to be feasible, acceptable, and effective by IBD patients. Further research on innovative, evidence-based, multidisciplinary educational interventions and the impact of these interventions on IBD clinical outcomes are needed.

**Funding Agencies:**

None

